# A New Mutual Information Estimator for Continuous Censored Variables

**DOI:** 10.3390/e28060677

**Published:** 2026-06-11

**Authors:** Ima Bernada, Cécilia Samieri, Grégory Nuel

**Affiliations:** 1 Bordeaux Population Health, Institut National de la Santé et de la Recherche Médicale, 33000 Bordeaux, France; 2 Laboratoire de Probabilités, Statistique et Modélisation, Institut National des Sciences Mathématiques et de Leurs Interactions, Centre National de la Recherche Scientifique, Sorbonne Université, 75005 Paris, France

**Keywords:** mutual information, censoring, estimation, simulations

## Abstract

Estimating dependency relationships between variables is an important issue in statistics. Mutual information (MI) is a measure of dependency which quantifies the amount of shared information between two variables. It is free of distribution assumption and captures both linear and non-linear dependencies. MI estimation methods were primarily developed for datasets with exclusively discrete variables, exclusively continuous variables, or a mixture of both. In practice, complex variables containing both discrete and continuous values (discrete-continuous variables), specifically continuous censored variables, are often present in real datasets (e.g., biological measures from analytical tools with lower detection limit). Methods have been developed to handle discrete-continuous data, but their effectiveness on the specific case of continuous censored data has not yet been evaluated. We propose a new estimation method based on the decomposition of the MI formula, with a first part handling the censoring status of the data, and a second part handling its continuous section. This estimation method works as a correction, as it takes in parameter one MI estimator for continuous data, and makes it able to handling censoring. We constructed different simulation scenarios of pairs of correlated censored log-normal variables, by varying the censoring rate, correlation, and sample size. We evaluated our correction on a few existing estimators previously developed for continuous, mixed or discrete-continuous data. We compared the selected estimators, with and without the correction, on these different scenarios. We found that the correction globally enables to reduce bias, and allows convergence towards the true MI value as the number of observations increases.

## 1. Introduction

Understanding and quantifying dependencies between variables is an important issue in statistics. Mutual information (MI) is a measure of dependency based on entropy [1,2], and corresponds to the quantity of shared information between two variables. It is largely used in network sciences [3,4,5], as it presents very interesting properties: it can be used without making hypothesis on the underlying distribution of the variables and is applicable on both discrete and continuous variables; and it allows capturing both linear and non-linear dependencies. Conditional mutual information (CMI) is also very interesting as it characterizes the dependency between two variables controlling for the other dependencies that may arise between each of these two variables and other variables in a dataset.

**Figure 1:**
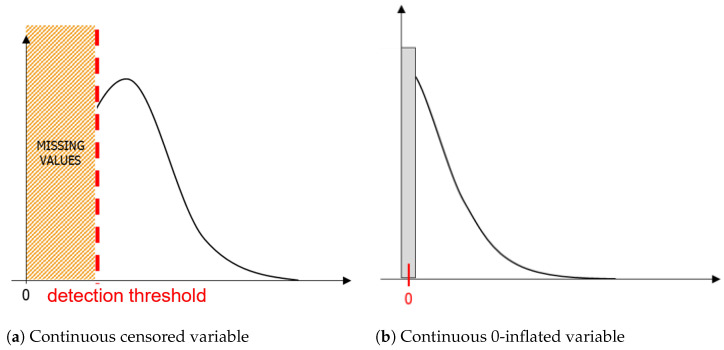
Two types of discrete-continuous variables.

Methods for estimating MI and CMI, denoted as (C)MI when referring to both information measures, were primarily developed for exclusively discrete or exclusively continuous variables [6,7,8,9]. But in practice, we may deal with mixed data, i.e., datasets that can include both discrete and continuous variables. This type of dataset is often encountered in cohort studies for example. Complex variables containing both discrete and continuous values (referred to as discrete-continuous variables) are also often present in cohort datasets. A common type of discrete-continuous variable is continuous censored. For example, left-censoring is very common on biological measurements from analytic tools with a lower limit of detection. Values falling below that threshold cannot be estimated, and are therefore missing on the left side of the distribution (Figure 1a). These missing values can be replaced by zero (or a lower-bound value, such as the threshold of detection value), which, when these low values are numerous, leads to continuous 0-inflated distributions (Figure 1b). There are multiple other examples of 0-inflated variables in cohort studies. For instance, when estimating daily alcohol intake, the data is theoretically continuous, but many people do not drink daily, resulting in a large proportion of zero in the corresponding variable. In this article, we make the following distinction: in what we refer to as “continuous censored” variables, 0-inflation is caused by censoring due to measurement limits; whereas in “continuous 0-inflated” variables, zeros are due to the real behavior of the data.

Standard MI estimation methods for continuous variables are based on histogram models [7,10,11,12] or k-nearest neighbors (k-NN) statistics [6,13,14,15]. Histogram models estimate the probability density function of the data, by discretizing continuous values on non-overlapping intervals while k-NN models use distance between observations. Some MI estimation methods based on histogram models and k-NN statistics have been optimized to handle mixed data; however, these approaches do not address the specific problem of discrete-continuous distributions, and their utilization with such data may raise statistical issues. Thus, for example, with continuous censored variables, adaptive histogram estimators discretize the entire data set, considering the censored part in the same way as continuous values [11]. Censored values can therefore be contained in the same discretization intervals as continuous values, which leads to lose information on censoring. With the standard k-NN approaches (for continuous variables), estimation of MI in presence of censoring is usually impossible because the distance between two neighbor values can be null. A few other methods have been developed to handle more specifically discrete-continuous data [12,13,14,15] but their effectiveness on the specific case of continuous censored data has not yet been demonstrated.

An intuitive approach to handle the censoring problem is to deconstruct the MI formula in order to handle the discrete status (either censoring or 0-inflation) on one side (considered as a binary variable), and continuous data on the other side. This second part can be estimated by any MI estimator adapted to continuous data.

In this paper, we propose a new MI estimator, based on a decomposition of continuous and censored/inflated parts of the variables. It uses both a standard MI estimator for continuous data, and empirical probabilities to estimate the MI of the censoring (or inflation) status. We evaluate our correction on a simple Gaussian approximation method (adapted for continuous data) and a few existing estimators previously developed for mixed or discrete-continuous data [11,15]. We begin by briefly reviewing existing methods for estimation of MI for discrete, continuous or mixed data as well as methods adapted to discrete-continuous variables. Next, we present our new MI estimation method for continuous censored variables (which also applies to the situation of 0-inflation). We then evaluate the approach on simulated data, and discuss the results.

## 2. Materials and Methods

### 2.1. State of the Art on Existing Mutual Information Estimators

In the case of discrete variables, (C)MI estimation is easily achieved by estimating empirical probabilities of the variables. In contrast, with continuous data, the number of combinations in a variable is usually too important to do so, and the variables densities are estimated, for example, using Gaussian approximation, k-NN or kernel estimation, or through data discretization, using histogram models.

The MI of two random continuous variables *X* and *Y*, with support set X and Y, is defined as:

(1) $$\mathrm{MI}\left(X,Y\right)=\int_{X}\int_{Y}f_{X,Y}\left(x,y\right)\log\left(\frac{f_{X,Y}\left(x,y\right)}{f_{X}\left(x\right)f_{Y}\left(y\right)}\right)dxdy$$

The CMI of continuous random variables *X* and *Y* conditioned by *Z*, with *Z* a single variable or a set of variables, is defined as:

(2) $$\mathrm{CMI}\left(X,Y|Z\right)=\int_{X}\int_{Y}\int_{Z}f_{X,Y,Z}\left(x,y,z\right)\log\left(\frac{f_{X,Y|Z}\left(x,y|z\right)}{f_{X|Z}\left(x|z\right)f_{Y|Z}\left(y|z\right)}\right)dxdydz$$

#### 2.1.1. Gaussian Approximation Approach

A simple method for estimating the (C)MI of continuous variables is to assume their distribution to be Gaussian. (C)MI of a multivariate Gaussian distribution is simplified by a formula depending only on the correlation matrix of the variables [2]. Let (X,Y,Z)∼N(μ,Σ). $\rho_{XY}$ is the correlation coefficient of *X* and *Y*, $\sigma_{Z}^{2}$ is the variance of *Z*, $\Sigma_{XZ}$ is the correlation sub-matrix of *X* and *Z*, $\Sigma_{YZ}$ is the correlation sub-matrix of *Y* and *Z*, and |Σ|=det(Σ).

(3) $$\mathrm{MI}\left(X,Y\right)=-\frac{1}{2}\log\left(1-\rho_{XY}^{2}\right)$$

(4) $$\mathrm{CMI}\left(X,Y|Z\right)=\frac{1}{2}\log\left(\frac{{\left|\Sigma \right|}_{XZ}{\left|\Sigma \right|}_{YZ}}{\sigma_{Z}^{2}\left|\Sigma \right|}\right)$$

(C)MI estimation can then be achieved by estimating correlation coefficients between variables. This estimation method assumes the Gaussian distribution of the data, and is therefore likely to be less efficient when applied on discrete-continuous variables.

#### 2.1.2. k-NN Approach

(C)MI estimation of continuous data can also be achieved using k-NN statistics [7]. This approach is based on a method initially constructed for entropy estimation [16]. It consists in estimating the density of each variable by taking into account the distance of each point to its *k*th nearest neighbor.

An extension of k-NN based MI estimator for mixed data and discrete-continuous variables has been proposed [13]. For these non-purely continuous data, there is some probability that multiple independent observations will be equal, so there is a non-zero probability that the k-NN distance is null for some points, which causes estimation issues. This estimation method [13] allows k to change for points whose k-NN distance is zero. The method was further expanded to incorporate conditioning [14]. A variation of this CMI estimator has also been suggested (hereby refered as kNN_dc method (MI estimation based on k-NN statistics, adapted for discrete-continuous data) [15]), improving performances on simulated discrete-continuous data.

#### 2.1.3. Kernel Density Approach

The joint and marginal densities can also be estimated using kernel density estimation, and (C)MI estimation is then deduced from it [6]. Similarly to the choice of bins in the histogram approach, the choice of bandwidth parameter is critical in kernel estimation, determining the smoothness of the density estimate. This estimation method can be computationally costly [17] and thus will not be considered in our research.

#### 2.1.4. Histogram Model Approach

It is common to reduce the number of value combinations from two continuous variables by transforming the variables into discrete ones, using histogram models, by separating the continuous values into non-overlapping intervals called bins. (C)MI estimation is then carried-out on binned data, through computation of empirical probabilities. The characteristics of the bins, i.e., their width and quantity, have a strong impact on the (C)MI estimation and must be chosen cautiously. Arbitrary discretization tends to under-estimate the real (C)MI value for small number of bins and to over-estimate it for large number of bins [11]. To overcome this problem, adaptive histogram models have been developed to optimize the size, number and position of the bins for each variable [18] in (C)MI estimation [11,12]. Adaptive histogram model approaches were subsequently improved to handle mixed data (hereby refered as AHist_mixed method (MI estimation using Adaptive Histogram model, adapted to mixed variables) [11]).

While complex methods have been developed to deal with discrete (repeated) values in continuous variables [11,12,13,14,15], it is also common to deal with these discrete values by adding a very low-amplitude noise to the data, and using a (C)MI estimator for continuous values. This method was first proposed to deal with the presence of repeated continuous values in real datasets [7], and can therefore be used for discrete-continuous data, and thus continuous censored data. However, it has been shown to perform worse than existing estimators constructed specifically for discrete-continuous data, on simulations, for MI estimation [13], and for CMI estimation [14], so it will not be considered in our research.

In this work, we focused on a few (C)MI estimation methods that appeared among the best ones based on current state-of-the-art literature to estimate (C)MI in presence of discrete-continuous data. We considered: (i) a simple Gaussian approximation as defined in Equation (4), and using Pearson correlation estimation; (ii) a k-NN method (kNN_dc) [15]; (iii) an adaptive histogram model-based method (AHist_mixed [11]). We decided to take an interest in these estimators as they are very diversified in terms of methodology and in their capacity to handle censoring.

We proposed a new correction for (C)MI estimators adapted for continuous censored variables, based on the theoretical results shown in Section 2.2, and evaluated the impact of the correction on these selected estimators using simulated data.

#### 2.1.5. Implementation of Used Estimation Methods

Methods relying on k-NN statistics and histogram models have been implemented by their authors, in R, for AHist_mixed [11] or Python, for kNN_dc [15]. We applied AHist_mixed using *discretizeMutual* function, available in R package **miic**; kNN_dc using *cmi* Python function; and Gaussian approximation using *cor* function from **stats** R package. AHist_mixed using *discretizeMutual* function is applied with and without the correction proposed by its authors to account for the finite size of the dataset (respectively AHistc_mixed and AHist_mixed) [11]. R was used in version 4.4.2 and Python in version 3.12.

We applied *discretizeMutual* using the default maxbins and cplx values, which correspond to the maximum number of bins desired in the discretization (by default $5\sqrt[{3}]{N}$ with *N* the number of observations) and the complexity used (by default the Normalized Maximum Likelihood complexity). For kNN_dc, we used k=7 as in the authors’ paper [15]. For Gaussian approximation, we used Pearson correlation estimation, using *cor* function’s “pearson” method parameter.

### 2.2. Correction of (C)MI Computation to Handle Censoring in the Context of Continuous Censored Variables

#### 2.2.1. Mutual Information for Continuous Censored Variables

Let *X* and *Y* be two random continuous variables, left-censored at threshold $\alpha_{X}$ and $\alpha_{Y}$ respectively. We assume that every missing value (due to censoring) is replaced by the variable’s threshold value. Let $X_{C}$ and $Y_{C}$ be binary variables representing the censoring status of *X* and *Y* respectively, such as

$$X_{C}=\left\{\begin{array}{cc} 1 & \;\mathrm{if}\;X>\alpha_{X}\\ 0 & \;\mathrm{if}\;X=\alpha_{X} \end{array}\right.\;\mathrm{and}\;Y_{C}=\left\{\begin{array}{cc} 1 & \;\mathrm{if}\;Y>\alpha_{Y}\\ 0 & \;\mathrm{if}\;Y=\alpha_{Y} \end{array}\right.$$

MI of *X* and *Y* can be defined as:

(5) $$\begin{array}{c} \mathrm{MI}\left(X,Y\right)=\mathrm{MI}\left(X_{C},Y_{C}\right)\\ +P\left(X>\alpha_{X},Y>\alpha_{Y}\right)\int_{\alpha_{X}}^{+\infty }\int_{\alpha_{Y}}^{+\infty }P\left(X=x,Y=y|X>\alpha_{X},Y>\alpha_{Y}\right)\log\left(\frac{P\left(X=x,Y=y|X>\alpha_{X},Y>\alpha_{Y}\right)}{P\left(X=x|X>\alpha_{X}\right)P\left(Y=y|Y>\alpha_{Y}\right)}\right)dxdy\\ =\mathrm{MI}\left(X_{C},Y_{C}\right)+P\left(X>\alpha_{X},Y>\alpha_{Y}\right)\mathrm{MI}\left(X,Y|X>\alpha_{X},Y>\alpha_{Y}\right) \end{array}$$

Theoretical developments are shown in the Appendix A.

In the case of CMI, with *Z* a variable censored to threshold $\alpha_{Z}$, and with $Z_{C}$ the censoring status of *Z*, we have

(6) $$\begin{array}{c} \mathrm{CMI}\left(X,Y|Z\right)=\mathrm{CMI}\left(X_{C},Y_{C}|Z_{C}\right)\\ +P\left(X>\alpha_{X},Y>\alpha_{Y},Z>\alpha_{Z}\right)\mathrm{CMI}\left(X,Y|Z,X>\alpha_{X},Y>\alpha_{Y},Z>\alpha_{Z}\right) \end{array}$$

Based on MI formula, specifically Equations (5) and (6), we developed a correction suitable for every (C)MI estimator existing for continuous variables, in order for them to handle censoring.

#### 2.2.2. Correction of (C)MI Computation to Handle Variable’s Censoring

The MI of two censored variables can be decomposed as in Equations (5) and (6). This corresponds to the addition of two terms, the first one corresponding to the MI of the censoring status of the variables, and the second one corresponding to the MI of the continuous part of the variables, weighted by the probability that the variables are simultaneously uncensored. Using these results of MI formula presented just above, we define MI estimation for two continuous censored variables as follows:

(7) $$\begin{array}{c} \hat{\mathrm{MI}}\left(X,Y\right)=\hat{\mathrm{MI}}\left(X_{C},Y_{C}\right)\\ +\hat{P}\left(X>\alpha_{X},Y>\alpha_{Y}\right)\hat{\mathrm{MI}}\left(X,Y|X>\alpha_{X},Y>\alpha_{Y}\right) \end{array}$$

The first part of the formula $\hat{\mathrm{MI}}\left(X_{C},Y_{C}\right)$, corresponding to the MI of the censoring status, and $\hat{P}\left(X>\alpha_{X},Y>\alpha_{Y}\right)$, the probability that the variables are simultaneously uncensored, are estimated by the contingency tables of the censoring status of the variables. The MI of simultaneously uncensored variables $\hat{\mathrm{MI}}\left(X,Y|X>\alpha_{X},Y>\alpha_{Y}\right)$ is estimated using a chosen MI estimation method adapted for continuous variables. This estimation does not require knowing the censoring threshold values of the variables. It requires the construction of a dataset containing only the simultaneously non-zero (or non-missing) values of the variables. The MI estimation method adapted for continuous variables is then applied to this dataset.

In the case of CMI with one conditional variable *Z*, the estimation formula is defined as:

(8) $$\begin{array}{c} \hat{\mathrm{CMI}}\left(X,Y|Z\right)=\hat{\mathrm{CMI}}\left(X_{C},Y_{C}|Z_{C}\right)\\ +\hat{P}\left(X>\alpha_{X},Y>\alpha_{Y},Z>\alpha_{Z}\right)\hat{\mathrm{CMI}}\left(X,Y|Z,X>\alpha_{X},Y>\alpha_{Y},Z>\alpha_{Z}\right) \end{array}$$

## 3. Results: Evaluation of the New (C)MI Estimator for Continuous Censored Variables on Simulated Data

In this section, we evaluated the impact of the (C)MI estimator correction on estimations obtained from a few selected estimators for continuous (Gaussian, Equation (4)), mixed [11], or discrete-continuous variables [15]. Simulated pairs or triplets of correlated variables were constructed, presenting a chosen rate of censoring or 0-inflation, as shown below (Experiment I–III). Supplementary results (Experiment IV–VI)) are shown in Supplementary File S1. We considered 0-inflated variables following a log-normal distribution with probability *p*, and equal to zero with probability 1−p. Continuous censored variables were constructed from continuous log-normal variables, censored under a chosen threshold.

To evaluate the performances of the estimators without and with the correction, on continuous 0-inflated or continuous censored data, we constructed different simulation scenarios, presented in Table 1 and Table S3. Each scenario was replicated 500 times.

(C)MI was computed from these simulated datasets using the selected estimators, with and without the proposed correction. We compared the different (C)MI estimation results with the theoretical value of (C)MI, calculated beforehand, in order to evaluate the performances of the correction on these estimators.

**Figure 2:**
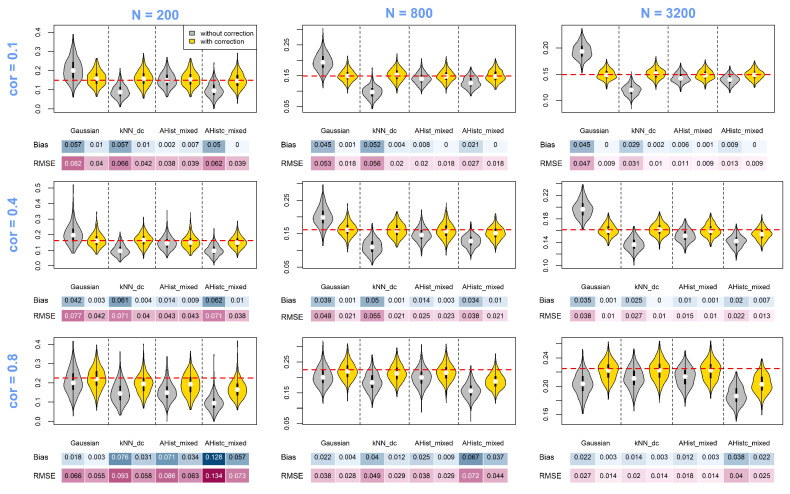
Two continuous 0-inflated variables (Experiment I). MI estimation for *X* and *Y* such as P(X=0,Y=0)=0.7,P(X≠0,Y=0)=0.05,P(X=0,Y≠0)=0.1,P(X≠0,Y≠0)=0.15. When not equal to zero, (X,Y)∼log−N(0,Σ) with $\Sigma =\left(\begin{array}{cc} 1 & \rho \\ \rho & 1 \end{array}\right)$. Rows correspond to correlation levels $\rho \in \left\{0.1,0.4,0.8\right\}$, and columns correspond to sample size $N\in \left\{200,800,3200\right\}$. Within each panel, each pair of violin plots represent one estimation method, with grey violin plots representing estimation without correction, and yellow ones with correction, while the red dashed line corresponds to the theoretical MI value. For every method, beneath the violin plots are displayed corresponding bias and RMSE values.

**Figure 3:**
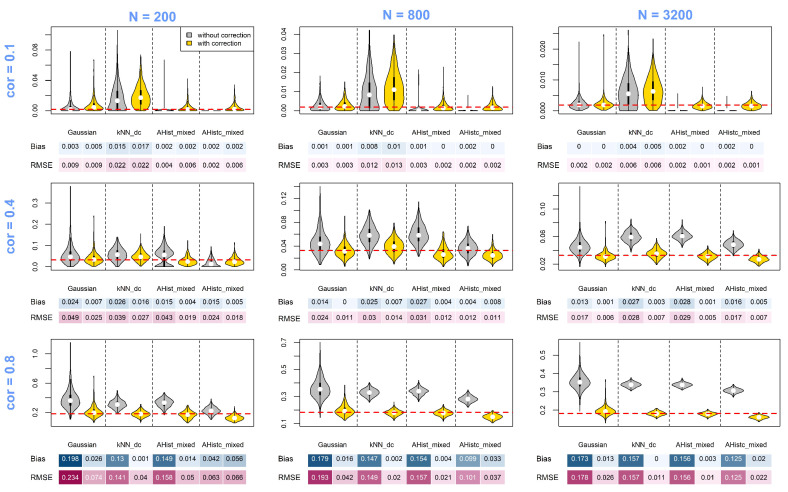
Two continuous censored variables (Experiment II. A). MI estimation for *X* and *Y* censored log-normal distributions—i.e., (X,Y)∼log−N(0,Σ), with $\Sigma =\left(\begin{array}{cc} 1 & \rho \\ \rho & 1 \end{array}\right)$. $P\left(X<\alpha_{X}\right)=P\left(X=0\right)=0.2$ and $P\left(Y<\alpha_{Y}\right)=P\left(Y=0\right)=0.6$. Rows correspond to correlation levels $\rho \in \left\{0.1,0.4,0.8\right\}$, and columns correspond to sample size $N\in \left\{200,800,3200\right\}$. Within each panel, each pair of violin plots represent one estimation method, with grey violin plots representing estimation without correction, and yellow ones with correction, while the red dashed line corresponds to the theoretical MI value. For every method, beneath the violin plots are displayed corresponding bias and RMSE values.

**Figure 4:**
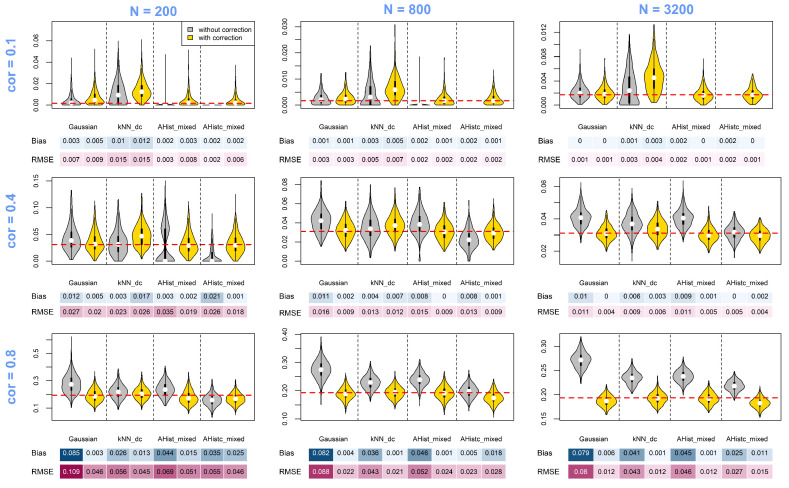
Two continuous censored variables (Experiment II. B). MI estimation for *X* and *Y* censored log-normal distributions—i.e., (X,Y)∼log−N(0,Σ), with $\Sigma =\left(\begin{array}{cc} 1 & \rho \\ \rho & 1 \end{array}\right)$. $P\left(X<\alpha_{X}\right)=P\left(X=0\right)=P\left(Y<\alpha_{Y}\right)=P\left(Y=0\right)=0.7$. Rows correspond to correlation levels $\rho \in \left\{0.1,0.4,0.8\right\}$, and columns correspond to sample size $N\in \left\{200,800,3200\right\}$. Within each panel, each pair of violin plots represent one estimation method, with grey violin plots representing estimation without correction, and yellow ones with correction, while the red dashed line corresponds to the theoretical MI value. For every method, beneath the violin plots are displayed corresponding bias and RMSE values.

**Figure 5:**
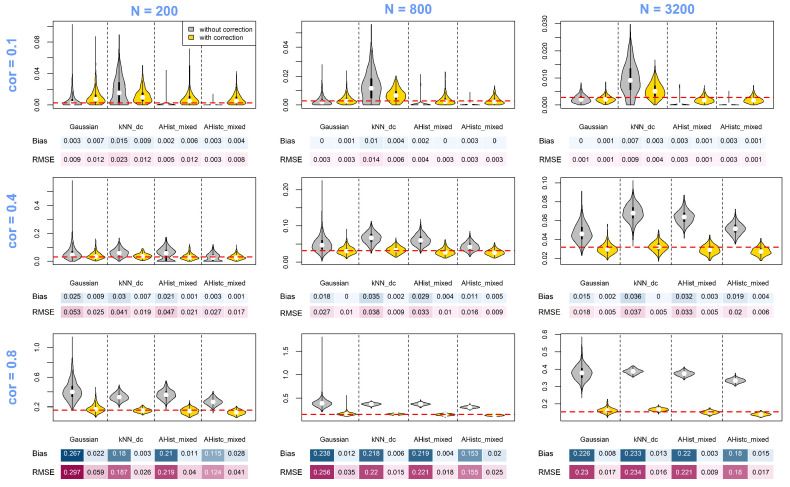
Two continuous censored variables and a third dependent continuous censored variable (Experiment III). CMI estimation for *X*, *Y* and *Z* censored log-normal distributions—i.e., (X,Y,Z)∼log−N(0,Σ), with $\Sigma =\left(\begin{array}{ccc} 1 & \rho & 0.3\\ \rho & 1 & 0\\ 0.3 & 0 & 1 \end{array}\right)$. $P\left(X<\alpha_{X}\right)=P\left(X=0\right)=0.2$, $P\left(Y<\alpha_{Y}\right)=P\left(Y=0\right)=0.6$, $P\left(Z<\alpha_{Z}\right)=P\left(Z=0\right)=0.5$. Rows correspond to correlation levels $\rho \in \left\{0.1,0.4,0.8\right\}$, and columns correspond to sample size $N\in \left\{200,800,3200\right\}$. Within each panel, each pair of violin plots represent one estimation method, with grey violin plots representing estimation without correction, and yellow ones with correction, while the red dashed line corresponds to the theoretical CMI value. For every method, beneath the violin plots are displayed corresponding bias and RMSE values.

The results are presented in the figures below (Figure 2, Figure 3, Figure 4 and Figure 5). Every sub-figure represents the results, in the form of violin plots, showing both the trend and the density of the estimates, for the four selected (C)MI estimators, both without the correction (in grey) and with the correction (in yellow). Theoretical MI values are displayed as a red dashed line. Bellow each estimator’s violin plot is displayed the corresponding bias and RMSE values, with cell color intensity reflecting estimation quality, with darker colors indicating less accurate estimations.

We first evaluated our correction under the situation of continuous 0-inflated variables (Experiment I, Figure 2). Without the correction (grey violin plots), whatever the sample size and pairwise correlation level, AHist_mixed, AHistc_mixed and kNN_dc tended to underestimate the MI of two inflated variables, while Gaussian tended to overestimate it. In all situations, the median of the estimates obtained with the correction (yellow violin plots) were improved compared to estimates obtained without correction, and the obtained values overlapped with the theoretical MI value.

When we estimated the performance of our correction under the scenario of continuous censored variables (Experiment II. A, Figure 3), we found that for two variables censored at 60% and 20%, the use of the correction generally reduced estimation bias. For small correlations (ρ=0.1, top line), the improvement in the estimation with the correction was marginal. The correction improved the underestimation of MI found with AHist_mixed and AHistc_mixed, but did not improve kNN_dc (bias increased with correction, for example from 0.015 to 0.017 when N=200, while variance decreased). Improvement was more obvious as the correlation reached 0.4 and above, for all estimators, and was also improved by the increase in the sample size.

When conditioning on a third continuous censored variable (Experiment III, Figure 5), results were similar.

When taking an interest in continuous censored variables presenting high and equal censoring rate (Experiment II. B, Figure 4), we found that the correction did not improve performances of kNN_dc when the correlation is small (ρ=0.1, top line). For higher correlation values, the correction improved the overestimation of Gaussian, kNN_dc, AHist_mixed and AHistc_mixed. For all estimation methods, but especially Gaussian, and correlation equal to 0.4 and above, the correction also enabled a reduction of the results variability.

Results of a representative setting, with average sample size (N=800) and correlation magnitude (ρ=0.4) when compared to all tested settings, are provided in Supplementary File S1 to summarize overall estimation performance across experiments. Overall, the correction led to more substantial improvements for continuous zero-inflated variables (Experiment I), where estimators tended to underestimate MI.

In summary, the results across the different experiments and settings indicate that the proposed correction largely improves (C)MI estimation in the case of continuous 0-inflated or continuous censored variables. However, for some estimators and under specific configurations, the correction showed no improvement. Even though the correction is applicable to any (C)MI estimator for continuous variables, the results observed in this study suggest that its relevance depends on both the data (sample size, censoring rates, correlation) and the estimation method used. Based on these results, recommendations regarding the use of the correction under different scenarios are summarized in Table 2.

Supplementary results showing results of estimation for pairs of variables of different types (e.g., one continuous censored and one continuous uncensored) are presented in Supplementary File S1.

Computational time was assessed for all experiments, estimation methods and settings (values of correlation and sample size). Applying the correction in the most complex scenario (one conditioning variable, Experiment III), made computational time increase of maximum 0.045 s, which remains reasonable (Table 2, Supplementary File S1). Overall, applying the correction slightly increased computational time for Gaussian, AHist_mixed and AHistc_mixed in most settings, while reducing it for kNN_dc, likely due to the reduced sample size after removing censored observations. Computational time remains to be evaluated in more complex scenarios.

## 4. Discussion

In this paper, we proposed a correction method for existing (C)MI estimators adapted to continuous data, in order to make them robust to inflation and censoring. This correction is based on the two-part decomposition of the (C)MI formula, the first part dealing with the inflation/censoring status of the variables values using empirical probabilities, and the second part dealing with the continuous values of the variables, using any (C)MI estimator able to handle continuous data.

This correction, although built for continuous inflated/censored data, can be applied to any continuous data, whether it presents inflation/censoring or not. The inflation/censoring status component of the decomposed (C)MI formula equals zero when the data is purely continuous. Since the correction term cancels out, the estimate corresponds to that made by the chosen (C)MI estimator for continuous variables. Thanks to this simplification, the correction remains applicable on a dataset containing both purely continuous and continuous inflated/censored variables.

We selected a group of estimators, based on their performances, estimation method, and diverse complexity levels (a Gaussian approximation method, a k-NN approach [15] and histogram models [11]). We then evaluated the correction method on these estimators by calculating their (C)MI estimation, both with and without the correction, using simulated pairs of continuous 0-inflated or continuous censored variables. The results showed meaningfully improved performance with the correction, compared to without. The correction was generally able to bring the median of the estimates closer to the theoretical (C)MI value, and sometimes reduced the estimates variance. Among the tested estimators, we took an interest in one (C)MI estimators constructed for discrete-continuous variables [15], a category of variables including continuous 0-inflated and continuous censored variables as defined in the article. We showed that for this estimator, as well as for the others tested, less suited to managing inflated/censored data, using this correction enables to better characterize (C)MI for variables containing inflation or censoring, often encountered in real datasets. However, performances of the correction are dependent on many parameters, such as the sample size, correlation between variables, censoring rate of variables, as well as the estimation method. For example, in scenarios where sample size is small, and correlation is low, we observed poorer performances of the correction across most estimation methods. Under these conditions, it is probable that the (C)MI of the simultaneously continuous parts of the variables is estimated with lower accuracy, whereas the (C)MI associated with the censoring status remains relatively robust. This is likely due to limited available information, as the proportion of data that is simultaneously continuous for all variables becomes very small. This would explain why we observed a more limited improvement with our correction in strong-censoring and low-correlation scenarios.

Although we have tested our approach under a scenario with one conditional variable, the next steps of this study would be to adapt this method to a more general conditional case. A current extension of this work aims at implementing a generalized version of the proposed correction, to make it applicable to information measures involving larger number of variables. This extension will enable the evaluation of the correction on datasets containing more than three variables. In particular, the generalized correction is intended to be evaluated in the context of graph reconstruction algorithms, therefore avoiding the theoretical computation of CMI with multiple conditioning variables. Computational cost will also be re-evaluated in this generalized framework in order to assess the impact of the number of conditioning variables on estimation time.

However, it is likely that using CMI estimation formula (Equation (8)) would be less effective for a large number of conditional variables presenting inflation or censoring. As the number of conditional variables increases, the number of simultaneously continuous variables values decreases, and the estimation of the continuous part of the data becomes less robust. Therefore, the use of the correction becomes less efficient. Moreover, the contingency tables, allowing to obtain the estimates of the joint probabilities of inflation or censoring status of the variables, would be very large, and the estimate would therefore not be as accurate as the estimate for few/no variables in conditioning, for the same sample size. The method would also be quite costly, as it requires knowing all the combinations of censoring (or inflation) status of the variables to estimate the joint status probabilities. This issue could therefore also be addressed in future research.

In this study, we assume that the non-censored component of the data is absolutely continuous, i.e., it contains no ties within each variable. We also do not account for the truncated nature of the continuous distributions induced by censoring, which may introduce boundary effects and potentially affect the performance of continuous estimators. Further work could investigate improved estimation procedures for this truncated continuous component, in order to better account for these effects.

Even though the simulations presented in this article are made using underlying log-normal distributions, we believe that the efficiency of the correction would be similar on other data distributions, as well as for variables presenting non-linear relationships. Indeed, theoretically and by definition, the performances of the correction are directly related to that of the used (C)MI estimator for continuous variables. Even though the correction adds an inflation/censoring status term to the formula, the continuous part of the data is calculated only using a chosen preexisting (C)MI estimator.

To the best of our knowledge, this correction is the first (C)MI estimation tool, adapted to continuous 0-inflated and continuous censored data, able to reduce the (C)MI estimation bias for these variables. These continuous 0-inflated and continuous censored data are extremely common in epidemiology and biological science datasets, and we believe that the correction we propose is essential for researchers to show true associations.

## Figures and Tables

**Table 1:** Simulation scenarios.

Experiment I	Two continuous 0-inflated variables	(X,Y)∼0-inflated log−N(0,Σ) i.e., their distribution is determined by the probabilities P(X=0,Y=0)=0.7,P(X≠0,Y=0)=0.05,P(X=0,Y≠0)=0.1,P(X≠0,Y≠0)=0.15. When not equal to zero, (X,Y)∼log−N(0,Σ) with $\Sigma =\left(\begin{array}{cc} 1 & \rho \\ \rho & 1 \end{array}\right)$.	Variation of sample size (N∈{200,800,3200}); variation of correlation of log-normal distributions (ρ∈{0.1,0.4,0.8})
Experiment II	Two continuous censored variables	(X,Y)∼log−N(0,Σ), with $\Sigma =\left(\begin{array}{cc} 1 & \rho \\ \rho & 1 \end{array}\right)$. *X* is left-censored at a threshold $\alpha_{X}$, *Y* is left-censored at a threshold $\alpha_{Y}$. All censored values are put to 0. Experiment II. A: $P\left(X<\alpha_{X}\right)=P\left(X=0\right)=0.2$, $P\left(Y<\alpha_{Y}\right)=P\left(Y=0\right)=0.6$ Experiment II. B: $P\left(X<\alpha_{X}\right)=P\left(X=0\right)=P\left(Y<\alpha_{Y}\right)=P\left(Y=0\right)=0.7$	
Experiment III	Two continuous censored variables and a third dependent continuous censored variable (for conditioning)	(X,Y,Z)∼log−N(0,Σ), with $\Sigma =\left(\begin{array}{ccc} 1 & \rho & 0.3\\ \rho & 1 & 0\\ 0.3 & 0 & 1 \end{array}\right)$. *X* is left-censored at a threshold $\alpha_{X}$, *Y* is left-censored at a threshold $\alpha_{Y}$, *Z* is left-censored at a threshold $\alpha_{Z}$. All censored values are put to 0. $P\left(X<\alpha_{X}\right)=P\left(X=0\right)=0.2$, $P\left(Y<\alpha_{Y}\right)=P\left(Y=0\right)=0.6$, $P\left(Z<\alpha_{Z}\right)=P\left(Z=0\right)=0.5$.	

**Table 2:** Recommendations for the use of the correction for unbiased estimation of (C)MI for continuous 0-inflated and continuous censored variables, for all estimation methods, experiments and settings tested. ✓ indicate scenarios where the correction improves (C)MI estimation; ✕ indicates scenarios where the correction does not improve (C)MI estimation; and Δ indicates scenarios where the performances of the correction depends on further context information (lightly censored conditioning variables, exact value of correlation, etc.).

Censoring Process	Correlation	Sample Size	Gaussian	kNN_dc	AHist_mixed	AHistc_mixed
Inflation, different inflation rates	Low	Small	✓	✓	✓	✓
		Large	✓	✓	✓	✓
	Medium to high	Small	✓	✓	✓	✓
		Large	✓	✓	✓	✓
Censoring, different censoring rates	Low	Small	Δ	Δ	✓	✓
		Large	✓	Δ	✓	✓
	Medium to high	Small	✓	✓	✓	Δ
		Large	✓	✓	✓	✓
Censoring, same censoring rates	Low	Small	✕	✕	✓	✓
		Large	✓	✕	✓	✓
	Medium to high	Small	✓	Δ	✓	Δ
		Large	✓	✓	✓	Δ

## Data Availability

The original data presented in the study are openly available in Zenodo, at https://doi.org/10.5281/zenodo.17367011.

## Supplementary Materials

The following supporting information can be downloaded at: https://www.mdpi.com/article/10.3390/e28060677/s1, Supplementary File S1: Supplementary results.

## Appendix A. Mutual Information Formulas

**Theorem** **A1** (MI for *X* continuous censored (or inflated) and *Y* continuous censored (or inflated)). *Let X and Y be two random continuous variable, left-censored at threshold $\alpha_{X}$ and $\alpha_{Y}$ respectively. In this development, we assume that every missing (censored) value is replaced by the variable’s threshold value (as it could be replaced by zero). MI is defined as:*

$$\begin{array}{c} \mathrm{MI}\left(X,Y\right)=\int_{X}\int_{Y}P\left(X=x,Y=y\right)\log\left(\frac{P\left(X=x,Y=y\right)}{P\left(X=x\right)P\left(Y=y\right)}\right)dxdy\\ =\int_{-\infty }^{\alpha_{X}}\int_{-\infty }^{\alpha_{Y}}P\left(X=x,Y=y\right)\log\left(\frac{P\left(X=x,Y=y\right)}{P\left(X=x\right)P\left(Y=y\right)}\right)dxdy\\ +\int_{-\infty }^{\alpha_{X}}\int_{\alpha_{Y}}^{\infty }P\left(X=x,Y=y\right)\log\left(\frac{P\left(X=x,Y=y\right)}{P\left(X=x\right)P\left(Y=y\right)}\right)dxdy\\ +\int_{\alpha_{X}}^{\infty }\int_{-\infty }^{\alpha_{Y}}P\left(X=x,Y=y\right)\log\left(\frac{P\left(X=x,Y=y\right)}{P\left(X=x\right)P\left(Y=y\right)}\right)dxdy\\ +\int_{\alpha_{X}}^{\infty }\int_{\alpha_{Y}}^{\infty }P\left(X=x,Y=y\right)\log\left(\frac{P\left(X=x,Y=y\right)}{P\left(X=x\right)P\left(Y=y\right)}\right)dxdy \end{array}$$

$$P\left(X=x,Y=y\right)=\left\{\begin{array}{cc} P\left(X=\alpha_{X},Y=\alpha_{Y}\right) & \mathrm{if}\;x=\alpha_{X}\;\mathrm{and}\;y=\alpha_{Y},\\ P\left(Y=y|Y>\alpha_{Y}\right)P\left(X=\alpha_{X},Y>\alpha_{Y}\right) & \mathrm{if}\;x=\alpha_{X}\;\mathrm{and}\;y>\alpha_{Y},\\ P\left(X=x|X>\alpha_{X}\right)P\left(X>\alpha_{X},Y=\alpha_{Y}\right) & \mathrm{if}\;x>\alpha_{X}\;\mathrm{and}\;y=\alpha_{Y},\\ P\left(X=x,Y=y|X>\alpha_{X},Y>\alpha_{Y}\right)P\left(X>\alpha_{X},Y>\alpha_{Y}\right) & \mathrm{if}\;x>\alpha_{X}\;\mathrm{and}\;y>\alpha_{Y} \end{array}\right.$$

$$\begin{array}{c} \mathrm{MI}\left(X,Y\right)=P\left(X=\alpha_{X},Y=\alpha_{Y}\right)\log\left(\frac{P\left(X=\alpha_{X},Y=\alpha_{Y}\right)}{P\left(X=\alpha_{X}\right)P\left(Y=\alpha_{Y}\right)}\right)\\ +\int_{\alpha_{Y}}^{+\infty }P\left(X=\alpha_{X},Y>\alpha_{Y}\right)P\left(Y=y|Y>\alpha_{Y}\right)\log\left(\frac{P\left(X=\alpha_{X},Y>\alpha_{Y}\right)}{P\left(X=\alpha_{X}\right)P\left(Y>\alpha_{Y}\right)}\right)dy\\ +\int_{\alpha_{X}}^{+\infty }P\left(X>\alpha_{X},Y=\alpha_{Y}\right)P\left(X=x|X>\alpha_{X}\right)\log\left(\frac{P\left(X>\alpha_{X},Y=\alpha_{Y}\right)}{P\left(X>\alpha_{X}\right)P\left(Y=\alpha_{Y}\right)}\right)dx\\ +\int_{\alpha_{X}}^{+\infty }\int_{\alpha_{Y}}^{+\infty }P\left(X>\alpha_{X},Y>\alpha_{Y}\right)P\left(X=x,Y=y|X>\alpha_{X},Y>\alpha_{Y}\right)\\ \times \log\left(\frac{P\left(X>\alpha_{X},Y>\alpha_{Y}\right)P\left(X=x,Y=y|X>\alpha_{X},Y>\alpha_{Y}\right)}{P\left(X>\alpha_{X}\right)P\left(Y>\alpha_{Y}\right)P\left(X=x|X>\alpha_{X}\right)P\left(Y=y|Y>\alpha_{Y}\right)}\right)dxdy\\ =P\left(X=\alpha_{X},Y=\alpha_{Y}\right)\log\left(\frac{P\left(X=\alpha_{X},Y=\alpha_{Y}\right)}{P\left(X=\alpha_{X}\right)P\left(Y=\alpha_{Y}\right)}\right)\\ +P\left(X=\alpha_{X},Y>\alpha_{Y}\right)\log\left(\frac{P\left(X=\alpha_{X},Y>\alpha_{Y}\right)}{P\left(X=\alpha_{X}\right)P\left(Y>\alpha_{Y}\right)}\right)\\ +P\left(X>\alpha_{X},Y=\alpha_{Y}\right)\log\left(\frac{P\left(X>\alpha_{X},Y=\alpha_{Y}\right)}{P\left(X>\alpha_{X}\right)P\left(Y=\alpha_{Y}\right)}\right)\\ +P\left(X>\alpha_{X},Y>\alpha_{Y}\right)\log\left(\frac{P\left(X>\alpha_{X},Y>\alpha_{Y}\right)}{P\left(X>\alpha_{X}\right)P\left(Y>\alpha_{Y}\right)}\right)\\ +P\left(X>\alpha_{X},Y>\alpha_{Y}\right)\int_{\alpha_{X}}^{+\infty }\int_{\alpha_{Y}}^{+\infty }P\left(X=x,Y=y|X>\alpha_{X},Y>\alpha_{Y}\right)\log\left(\frac{P\left(X=x,Y=y|X>\alpha_{X},Y>\alpha_{Y}\right)}{P\left(X=x|X>\alpha_{X}\right)P\left(Y=y|Y>\alpha_{Y}\right)}\right)dxdy\\ =\mathrm{MI}\left(X_{C},Y_{C}\right)+P\left(X>\alpha_{X},Y>\alpha_{Y}\right)\mathrm{MI}\left(X,Y|X>\alpha_{X},Y>\alpha_{Y}\right) \end{array}$$

**Theorem** **A2** (MI for *X* continuous censored (or inflated) and *Y* continuous). *Let X be a random continuous variable, left-censored at threshold $\alpha_{X}$. We assume that every missing (censored) value is replaced by the variable’s threshold value. Let Y be a random continuous (non-censored nor inflated) variable. MI is defined as:*

$$\begin{array}{c} \mathrm{MI}\left(X,Y\right)=\int_{-\infty }^{+\infty }\int_{-\infty }^{+\infty }P\left(X=x,Y=y\right)\log\frac{P(X=x,Y=y)}{P(X=x)P(Y=y)}dxdy\\ =\int_{-\infty }^{+\infty }P\left(X=\alpha_{X},Y=y\right)\log\frac{P(X=\alpha_{X},Y=y)}{P\left(X=\alpha_{X}\right)P\left(Y=y\right)}dy+\int_{-\infty }^{+\infty }\int_{\alpha_{X}}^{+\infty }P\left(X=x,Y=y\right)\log\frac{P(X=x,Y=y)}{P(X=x)P(Y=y)}dxdy\\ =\int_{-\infty }^{+\infty }P\left(X=\alpha_{X}\right)P\left(Y=y\right)\log\frac{P\left(X=\alpha_{X}\right)P\left(Y=y\right)}{P\left(X=\alpha_{X}\right)P\left(Y=y\right)}dy\\ +\int_{-\infty }^{+\infty }\int_{\alpha_{X}}^{+\infty }P\left(X=x,Y=y|X>\alpha_{X}\right)P\left(X>\alpha_{X}\right)\log\frac{P(X=x,Y=y|X>\alpha_{X})}{P\left(X=x|X>\alpha_{X}\right)P\left(Y=y\right)}dxdy\\ =P\left(X>\alpha_{X}\right)\int_{-\infty }^{+\infty }\int_{\alpha_{X}}^{+\infty }P\left(X=x,Y=y|X>\alpha_{X}\right)\log\frac{P(Y=y,X=x|X>\alpha_{X})}{P\left(X=x|X>\alpha_{X}\right)P\left(Y=y\right)}dxdy\\ =P\left(X>\alpha_{X}\right)\mathrm{MI}\left(X,Y|X>\alpha_{X}\right) \end{array}$$
