# Supplementary material for: A New Mutual Information Estimator for Continuous Censored Variables
Source: Entropy (Basel). 2026 Jun 11;28(6):677. doi: 10.3390/e28060677 (PMC13297969; doi:10.3390/e28060677)
Supplement: Supplementary file 1 [file entropy-28-00677-s001.zip › entropy-4188896-supplementary.pdf]

Article

Supplementary File S1: Supplementary results

Ima Bernada[1,*], Cécilia Samieri [1] and Grégory Nuel [2]

1 Bordeaux Population Health, Institut national de la santé et de la recherche médicale, 33000 Bordeaux, France
2 Laboratoire de probabilités, statistique et modélisation, Institut national des sciences mathématiques et de leurs interactions, Centre national de la recherche scientifique, Sorbonne Université, 75005 Paris, France
* Correspondence: ima.bernada@u-bordeaux.fr

1. Supplementary results of main experiments

(a) bias

	gaussian		kNN_dc		AHist_mixed		AHistc_mixed	
Experiment	nc	c	nc	c	nc	c	nc	c
Experiment I	0.0388	0.0011	-0.0501	-0.0012	-0.0145	-0.0025	-0.0337	-0.0098
Experiment II. A	0.0169	-0.0004	0.0342	0.0012	0.0287	-0.0042	0.0105	-0.0056
Experiment II. B	0.0110	0.0015	0.0037	0.0067	0.0082	0.0001	-0.0080	-0.0011
Experiment III	0.0177	0.0003	0.0349	0.0019	0.0294	-0.0035	0.0113	-0.0049

(b) RMSE

	gaussian		kNN_dc		AHist_mixed		AHistc_mixed	
Experiment	nc	c	nc	c	nc	c	nc	c
Experiment I	0.0488	0.0206	0.0547	0.0208	0.0254	0.0229	0.0380	0.0211
Experiment II A	0.0264	0.0100	0.0373	0.0092	0.0321	0.0102	0.0154	0.0096
Experiment II B	0.0159	0.0094	0.0128	0.0124	0.0150	0.0094	0.0128	0.0085
Experiment III	0.0269	0.0100	0.0380	0.0093	0.0327	0.0100	0.0159	0.0092

Table S1. Bias and RMSE values under all experiments (I, II.A, II.B, III), for key settings ($N = 800$ and $\rho = 0.4$). Each two columns show bias and RMSE values for each estimation method, without correction ('nc') and with correction ('c').

		gaussian		kNN_dc		AHist(c)_mixed	
Correlation	Sample size	nc	c	nc	c	nc	c
	200	< 0.001	0.002	0.006	0.017	0.004	0.004
0.1	800	< 0.001	0.009	0.049	0.063	0.022	0.014
	3200	0.001	0.046	0.606	0.217	0.042	0.079
	200	< 0.001	0.003	0.006	0.017	0.011	0.010
0.4	800	< 0.001	0.009	0.045	0.055	0.027	0.023
	3200	0.002	0.041	0.602	0.227	0.065	0.060
	200	< 0.001	0.003	0.006	0.017	0.027	0.005
0.8	800	< 0.001	0.010	0.045	0.055	0.025	0.017
	3200	0.001	0.042	0.629	0.214	0.057	0.074

Table S2. Computational time for Experiment III. For each estimation method, the two columns report runtimes without correction ('nc') and with correction ('c').

2. Supplementary experiments

Table S3. Simulation scenarios.

Experiment IV	One continuous and one continuous 0-inflated variable	$\mathbb{P}(X=0,Y=0)=0, \mathbb{P}(X\neq 0,Y=0)=0, P(X=0,Y\neq 0)=0.7, \mathbb{P}(X\neq 0,Y\neq 0)=0.3$. When not equal to zero, $(X,Y)\sim \log-\mathcal{N}(0,\Sigma)$ with $\Sigma=\begin{pmatrix}1 & \rho\\ \rho & 1\end{pmatrix}$	
Experiment V	One continuous and one continuous censored variable	$(X,Y)\sim \log-\mathcal{N}(0,\Sigma)$, with $\Sigma=\begin{pmatrix}1 & \rho\\ \rho & 1\end{pmatrix}$. Y is left-censored at a threshold α_Y. All censored values are put to 0. $\mathbb{P}(Y<\alpha_Y)=\mathbb{P}(Y=0)=0.4$	Variation of sample size ($N\in\{200,800,3200\}$); variation of correlation of log-normal distributions ($\rho\in\{0.1,0.4,0.8\}$)
Experiment VI	One continuous 0-inflated and one continuous censored variable	$Y=0$ with probability 0.45 and $(X,Y)\sim \log-\mathcal{N}(0,\Sigma)$, with $\Sigma=\begin{pmatrix}1 & \rho\\ \rho & 1\end{pmatrix}$. X is left-censored at a threshold α_X. All censored values are put to 0. $P(X<\alpha_X)=P(X=0)=0.25$.	

Under the three scenarios presented just above (Table S3), we found, as in main scenarios (Experiments I–III), that the correction enabled to reduce estimation bias in most cases. When we evaluated our correction under the scenario of one continuous 0-inflated and one continuous variable (Experiment IV, Figure S1), for pairwise correlation of $\rho=0.1$ (violin plots on the top row of the panel), whatever the sample size, we found a marginal improvement of the estimation with the correction, for all estimators. Similar results were found for one continuous censored and one continuous variable (Experiment V, Figure S2). In Figure S1 and Figure S2, for correlation greater than or equal to $\rho=0.4$, the estimations with the correction were better than the estimations without correction. The reduction in the variability of the estimations, linked to the increase in the size of the data set, strengthened these dissimilarities.

When we evaluated our correction on one continuous 0-inflated and one continuous censored variable (Experiment VI, Figure S3), we found that whatever the sample size and the pairwise correlation, estimations were better with the correction, for all estimators. The correction improved underestimation of MI found with Gaussian, AHist_mixed, AHistc_mixed, kNN_dc, and sometimes overestimation (depending on the correlation value) by kNN_dc. In this scenario, kNN_dc gives great estimation, only slightly improved by the correction in some cases. It is also the case in Experiment IV, for one continuous 0-inflated and one continuous variable (Figure S1), for pairwise correlation greater or equal to $\rho=0.4$. In Experiment VI (Figure S3), the correction also enabled to reduce variability of kNN_dc.

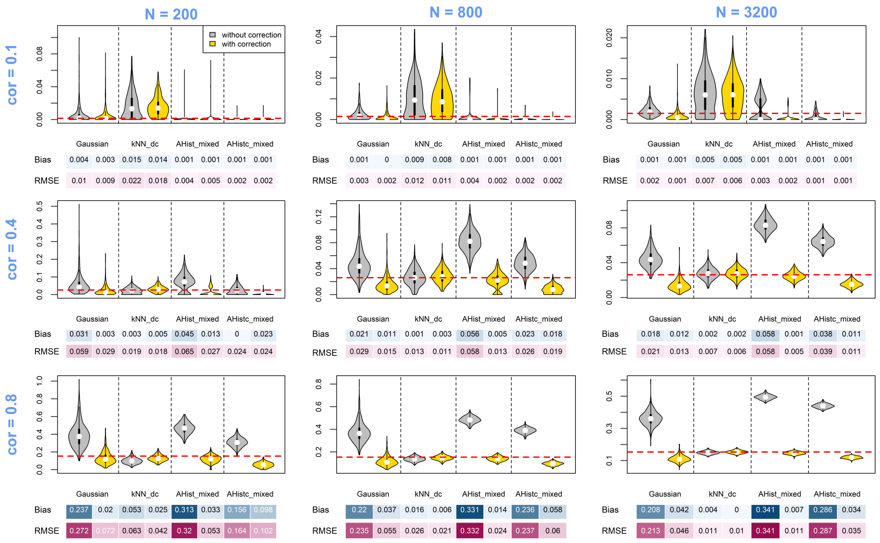

Figure S1. One continuous and one continuous 0-inflated variable (Experiment IV). MI estimation for X and Y respectively 0-inflated log-normal and log-normal distributions—i.e., $\mathbb{P}(X = 0, Y = 0) = 0, \mathbb{P}(X \neq 0, Y = 0) = 0, P(X = 0, Y \neq 0) = 0.7, \mathbb{P}(X \neq 0, Y \neq 0) = 0.3$. When not equal to zero, $(X, Y) \sim \log - \mathcal{N}(0, \Sigma)$ with $\Sigma = \begin{pmatrix} 1 & \rho \\ \rho & 1 \end{pmatrix}$. Rows correspond to correlation levels $\rho \in \{0.1, 0.4, 0.8\}$, and columns correspond to sample size $N \in \{200, 800, 3200\}$. Within each panel, each pair of violin plots represent one estimation method, with grey violin plots representing estimation without correction, and yellow ones with correction, while the red dashed line corresponds to the theoretical MI value. For every method, beneath the violin plots are displayed corresponding bias and RMSE values.

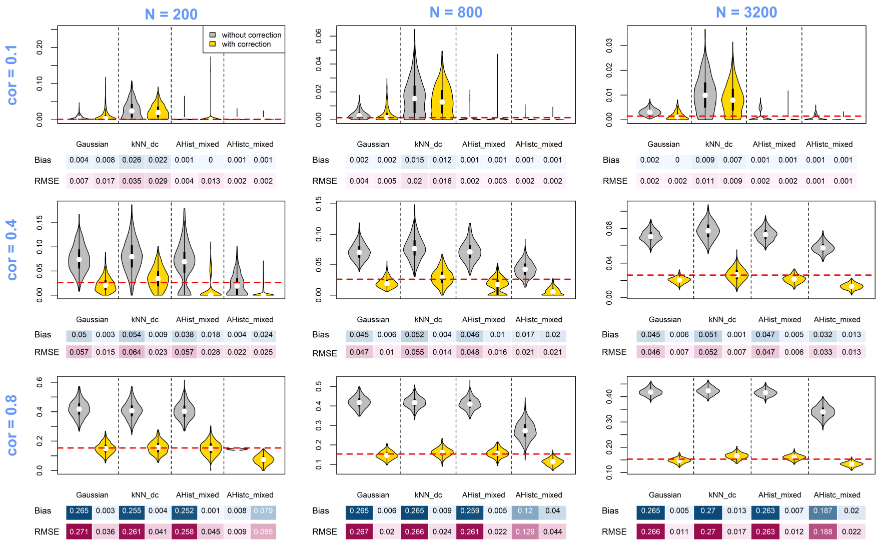

Figure S2. One continuous and one continuous censored variable (Experiment V). MI estimation for X and Y respectively censored and non-censored log-normal distributions—i.e., $(X, Y) \sim \log - \mathcal{N}(0, \Sigma)$, with $\Sigma = \begin{pmatrix} 1 & \rho \\ \rho & 1 \end{pmatrix}$. Y is left-censored at a threshold α_Y. All censored values are put to 0. $\mathbb{P}(Y < \alpha_Y) = \mathbb{P}(Y = 0) = 0.4$. Rows correspond to correlation levels $\rho \in \{0.1, 0.4, 0.8\}$, and columns correspond to sample size $N \in \{200, 800, 3200\}$. Within each panel, each pair of violin plots represent one estimation method, with grey violin plots representing estimation without correction, and yellow ones with correction, while the red dashed line corresponds to the theoretical MI value. For every method, beneath the violin plots are displayed corresponding bias and RMSE values.

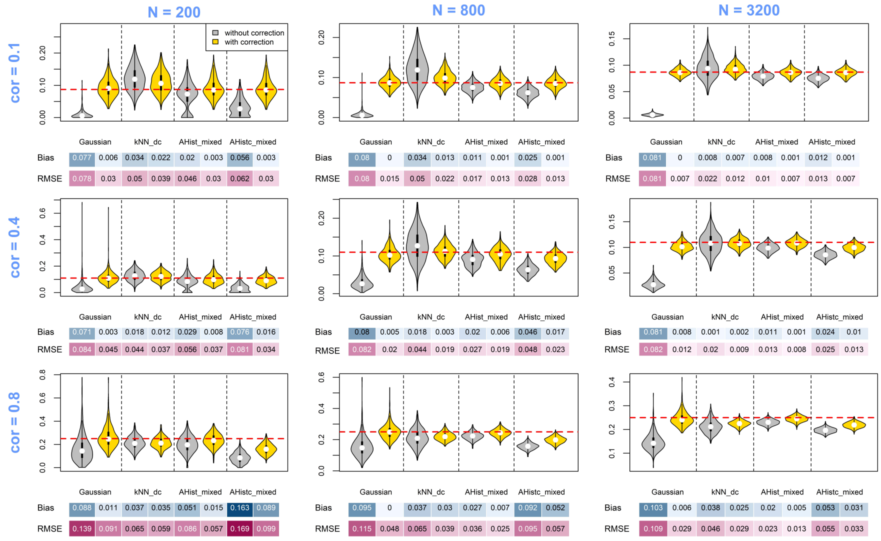

Figure S3. One continuous 0-inflated and one continuous censored variable (Experiment VI). MI estimation for X and Y respectively inflated and censored log-normal distributions—i.e., $Y = 0$ with probability 0.45 and $(X, Y) \sim \log -\mathcal{N}(0, \Sigma)$, with $\Sigma = \begin{pmatrix} 1 & \rho \\ \rho & 1 \end{pmatrix}$. X is left-censored at a threshold α_X. All censored values are put to 0. $P(X < \alpha_X) = P(X = 0) = 0.25$. Rows correspond to correlation levels $\rho \in \{0.1, 0.4, 0.8\}$, and columns correspond to sample size $N \in \{200, 800, 3200\}$. Within each panel, each pair of violin plots represent one estimation method, with grey violin plots representing estimation without correction, and yellow ones with correction, while the red dashed line corresponds to the theoretical CMI value. For every method, beneath the violin plots are displayed corresponding bias and RMSE values.

Disclaimer/Publisher's Note: The statements, opinions and data contained in all publications are solely those of the individual author(s) and contributor(s) and not of MDPI and/or the editor(s). MDPI and/or the editor(s) disclaim responsibility for any injury to people or property resulting from any ideas, methods, instructions or products referred to in the content.
